# Alcohol Consumption and Risk of Common Autoimmune Inflammatory Diseases—Evidence From a Large-Scale Genetic Analysis Totaling 1 Million Individuals

**DOI:** 10.3389/fgene.2021.687745

**Published:** 2021-06-22

**Authors:** Xia Jiang, Zhaozhong Zhu, Ali Manouchehrinia, Tomas Olsson, Lars Alfredsson, Ingrid Kockum

**Affiliations:** ^1^Department of Clinical Neuroscience, Center for Molecular Medicine, Karolinska Institute, Stockholm, Sweden; ^2^Department of Epidemiology, Harvard T.H. Chan School of Public Health, Boston, MA, United States

**Keywords:** Mendelian Randomization (MR), alcohol consumption amount, excessive drinking, autoimmune disease, genetic correlation, large-scale genetic analysis

## Abstract

**Purpose:** Observational studies have suggested a protective effect of alcohol intake with autoimmune disorders, which was not supported by Mendelian randomization (MR) analyses that used only a few (<20) instrumental variables.

**Methods:** We systemically interrogated a putative causal relationship between alcohol consumption and four common autoimmune disorders, using summary-level data from the largest genome-wide association study (GWAS) conducted on inflammatory bowel disease (IBD), rheumatoid arthritis (RA), multiple sclerosis (MS), and systemic lupus erythematosus (SLE). We quantified the genetic correlation to examine a shared genetic similarity. We constructed a strong instrument using 99 genetic variants associated with drinks per week and applied several two-sample MR methods. We additionally incorporated excessive drinking as reflected by alcohol use disorder identification test score.

**Results:** We observed a negatively shared genetic basis between alcohol intake and autoimmune disorders, although none was significant (*r*_*g*_ = −0.07 to −0.02). For most disorders, genetically predicted alcohol consumption was associated with a slightly (10–25%) decreased risk of onset, yet these associations were not significant. Meta-analyzing across RA, MS, and IBD, the three Th1-related disorders yielded to a marginally significantly reduced effect [OR = 0.70 (0.51–0.95), *P* = 0.02]. Excessive drinking did not appear to reduce the risk of autoimmune disorders.

**Conclusions:** With its greatly augmented sample size and substantially improved statistical power, our MR study does not convincingly support a beneficial role of alcohol consumption in each individual autoimmune disorder. Future studies may be designed to replicate our findings and to understand a causal effect on disease prognosis.

## Introduction

Alcohol contains components such as ethanol and antioxidants and is considered as a complex modulator to the immune system (Barr et al., 2016). Several *in vitro* and *in vivo* studies have demonstrated that ethanol modulates the function of monocytes and dendritic cells (innate immune cells) in a dose- and time-dependent manner. For example, while acute high-level exposure to ethanol inhibits proinflammatory cytokine production, long-term moderate administration of ethanol stimulates the process. In addition, *in vivo* consumption of moderate doses of alcohol enhances phagocytosis and reduces inflammatory cytokine production whereas chronic consumption of large doses inhibits phagocytosis and production of growth factors. For cell-mediated and humoral immunity (adaptive immunity), chronic alcohol abuse significantly reduces both the number and frequency of T lymphocytes, resulting in an increased proportion of memory T cells relative to naïve T cells, which interferes the development of efficacious responses to infection and vaccination. In contrast, moderate alcohol intake increases the frequency of lymphocytes. Moreover, alcohol also modulates the hypothalamic–pituitary–adrenal axis and influences the function of immune cells residing in the central nervous system (CNS) particularly astrocytes and microglia, which tightly regulates the stress response, neuronal function, and CNS homeostasis, in turn affecting immunity (Barr et al., 2016).

While it appears that high doses of alcohol directly suppress a wide range of immune responses and moderate doses of alcohol play a beneficial role in the immune system, the complex interplay among alcohol intake, immune response, and inflammatory processes remains to be understood (Romeo et al., 2007). The relationship between alcohol consumption and a number of chronic autoimmune inflammatory disorders has been investigated through conventional epidemiological studies, of which results remain inconclusive (Wang et al., 2008, 2015; Jin et al., 2014; Linneberg and Gonzalez-Quintela, 2016). It has been argued that the validity of findings from observational studies could be plagued by measurement error, confounding, and/or reverse causality.

Mendelian randomization (MR) is a novel statistical approach that uses genetic variants (instrumental variables, IVs; usually single-nucleotide polymorphisms, SNPs) as proxies to make causal inference between exposure(s) and outcome(s). Since genotypes are randomly assigned at conception and always precede disease onset, MR mirrors the randomization process in controlled trials and is less susceptible to confounding and reverse causality (Smith and Ebrahim, 2003). Nevertheless, application of MR in the field of autoimmune diseases remains limited—so far, only two MR(s) have been conducted to investigate the effect of alcohol with the risk of rheumatoid arthritis (RA; Bae and Lee, 2019b) and systemic lupus erythematosus (SLE; Bae and Lee, 2019a), each involving less than 20 genetic instruments.

A recent genome-wide association study (GWAS) conducted in alcohol drinking behavior (defined as drinks per week) has identified 99 significant independent loci (Liu et al., 2019), and the GWAS summary statistics for most autoimmune diseases have been made publicly available. Taking advantage of these enormous progresses made in genetic discoveries for complex traits, we aim to perform a large-scale comprehensive study to systemically interrogate the effect of alcohol consumption on a range of common autoimmune inflammatory disorders, leveraging the genetic information available for 1 million individuals of European ancestry. We will explore both a *shared genetic basis* as reflected by genetic correlation analysis and a *causal relationship* as reflected by MR analysis.

## Materials and Methods

We performed the current study employing a standard framework, that is, a genetic correlation analysis defined as the proportion of variance that two traits share due to genetic causes, and a two-sample MR analysis, where instrument–exposure (or IV–exposure, SNP–exposure) and instrument–outcome (or IV–outcome, SNP–outcome) associations were extracted from two independent non-overlapping sets of participants. For a conceptual framework of our MR (a flowchart of current study), please see Supplementary Figure 1; for characteristics of exposure and outcome genetic data, please see Supplementary Table 1.

### IV–Exposure

The hitherto largest GWAS of alcohol consumption was conducted using an imputation-accuracy-aware meta-analysis totaling 941,280 individuals of European ancestry recruited from 34 participating studies (Liu et al., 2019). The exposure, drinks per week, was defined as the average number of drinks a participant reported drinking each week, aggregated across all types of alcohol. If a participating study recorded binned response ranges (e.g., one to four drinks per week, 5–10 drinks per week), the midpoint of the range was used. The phenotype was left-anchored at 1 and log-transformed prior to analysis. This large-scale meta-GWAS has identified 99 genome-wide significant variants associated with drinks per week after conditional and joint analyses. We used these 99 independent SNPs as our instruments and extracted IV–exposure associations (beta-coefficients, standard errors) and relevant information (rsID, effect allele, allele frequency, genomic coordinates) from the abovementioned alcohol GWAS. Details on characteristics of the 99 IVs are presented in Supplementary Table 1. We also obtained full-set GWAS summary data for genetic correlation analysis.

While drinks per week reflect normal or general drinking behavior, we included one additional exposure, alcohol use disorder identification test consumption score (AUDIT), which reflects excessive or harmful drinking behavior. The GWAS of AUDIT was conducted in a multi-ancestry Million Veteran Program sample of 274,424 individuals, and 13 GWAS-significant independent loci were identified among Europeans to be associated with alcohol use disorder (Kranzler et al., 2019). We used these 13 SNPs as IVs to perform additional analysis and to complement with our main findings (Supplementary Table 2).

### IV-Outcome

We systemically examined the role of alcohol consumption in four autoimmune diseases. We collected the hitherto largest full-set GWAS summary data of inflammatory bowel disease (IBD; Liu et al., 2015) and its subsets [Crohn’s disease (CD) and ulcerative colitis (UC)], RA (Okada et al., 2014), SLE, (Bentham et al., 2015) and multiple sclerosis (MS; International Multiple Sclerosis Genetics Consortium, 2019), all of European ancestry. We selected these four autoimmune disorders due to two reasons: (1) they are common and (2) they had GWAS with decent sample size and SNP coverage (>5,000 cases and >10,000 controls and >1,000,000 genetic markers) to ensure statistical power. From these GWAS summary data, we extracted IV–outcome associations (beta-coefficients and standard errors) and relevant information (rsID, effect allele, allele frequency, genomic coordinates).

The abundant available samples make our study so far the largest of its kind, leveraging on the genetic information from 49,336 cases of autoimmune disorders and 108,387 controls (number of cases/controls for each outcome, IBD: 12,882/21,770; UC: 6,968/20,464; CD: 5,956/14,927; RA: 14,361/43,923; MS: 14,802/26,703; SLE: 7,291/15,991). Details of the outcome GWAS(s) are shown in Supplementary Table 3.

### Statistical Analysis

#### Genetic Correlation Analysis

The correlation between the genetic influences on a trait and the genetic influences on a different trait estimates the degree of *causal overlap or pleiotropy*. We quantified the genome-wide genetic correlation between alcohol consumption and each disorder, using an algorithm implemented in statistical software linkage disequilibrium score regression (LDSC). LDSC leverages the relationship between association statistics and linkage disequilibrium patterns across the genome and estimates the genetic correlation using only GWAS summary-level data (Bulik-Sullivan et al., 2015).

#### Mendelian Randomization Analysis

We next evaluated a *causal relationship* between alcohol consumption and autoimmune disorders. MR yields an unbiased causal estimate based on observational data only when three model assumptions are satisfied. Namely, IVs should be robustly associated with the exposure (relevance), affect outcome only through the exposure (exclusion restriction), and should not be associated with confounders in the exposure–outcome relationship (exchangeability). To guarantee model assumption, we applied several MR approaches including a random-effect inverse variance-weighted method (IVW; Burgess et al., 2015), a maximum likelihood approach (Burgess et al., 2013), a weighted median approach (Bowden et al., 2016), and an MR–Egger regression (Bowden et al., 2015).

Briefly, the random-effect IVW pools estimate from each IV and provide causal estimation, assuming that all IVs are valid or are invalid in a way that the overall pleiotropy is balanced to be zero (Burgess et al., 2015). When there is considerable imprecision in the estimates, causal effect estimates from the IVW are overprecise, whereas the likelihood method gives appropriately sized confidence intervals (Burgess et al., 2013). In addition, we performed MR–Egger regression to test for bias due to directional pleiotropy, where the average of direct effects of the tested genetic variants on outcome is non-zero (Bowden et al., 2015). We employed a weighted median to provide consistent estimates even when up to 50% of the analyzed genetic variants are invalid (Bowden et al., 2016).

In addition, we performed several important sensitivity analyses to further validify model assumptions. For example, we excluded palindromic IVs (SNPs with alleles represented by the same pair of letters on the forward and reverse strands such as A/T or G/C SNPs. These SNPs can introduce ambiguity into the identity of the effect allele in the exposure and outcome GWASs.) (Hemani et al., 2018). We excluded IVs that were associated with potential confounding traits according to the GWAS catalog. Further, we employed a multivariable MR approach to adjust for potential horizontal pleiotropy acting in particular through the body mass index and smoking—the two lifestyle behavioral traits tend to cluster together with alcohol consumption (Burgess and Thompson, 2015). We extracted IV-BMI effect sizes and IV-smoking effect sizes from the hitherto largest obesity (*N* = 700,000) (Yengo et al., 2018) and smoking (*N* = 1,232,091) (Liu et al., 2019) GWAS(s). Finally, we excluded one SNP at a time and performed IVW on the remaining SNPs to identify potential influence of outlying variants on the estimates.

Mendelian randomization methods evaluate an overall casual estimation; it is likely that several distinct causal mechanisms underlie the alcohol–disease relationship, in which a risk factor influences outcome with different magnitudes of causal effect. We examined such a scenario through MR-Clust (Foley et al., 2019), an approach that divides IVs into distinct clusters such that all variants in the cluster have similar causal estimates.

Finally, we complemented our main results of general drinking behavior, by incorporating genetic instruments associated with excessive or harmful drinking behavior (alcohol use disorder identification test). Given the fewer IVs associated with AUDIT (*N* = 13), we only performed primary analysis for this exposure (IVW and MR–Egger), as the diagnostic analyses including MVMR and MR-Clust were underpowered with the limited availability of genetic instruments.

We included four autoimmune disorders as main outcomes (CD and UC were treated as subsets of IBD) and performed analysis using different sets of instruments as well as different statistical approaches; our results were likely to suffer from false positives due to multiple comparisons. Therefore, we considered a two-sided P-threshold of 0.05 as suggestive significance. An arbitrarily corrected P-threshold of 0.01 (0.05/4) was used as statistical significance. All MR analyses were performed using R software version 4.0.2 with packages “TwoSampleMR,” “MendelianRandomization,” and “MRclust.”

## Results

**FIGURE 1:**
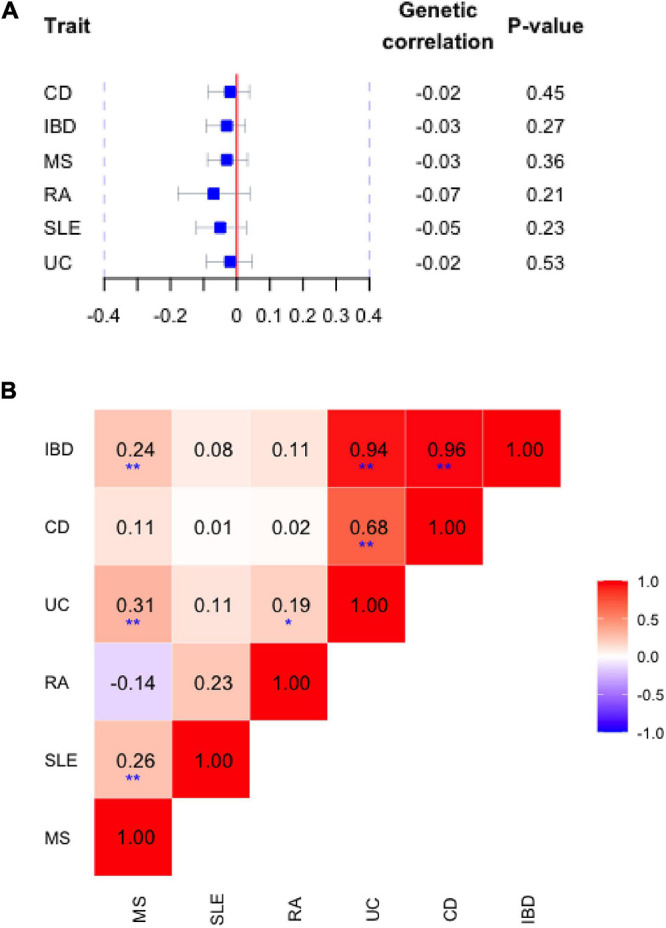
**(A)** Genetic correlation between alcohol consumption (drinks per week) and risk of autoimmune inflammatory diseases. Blue squares and horizontal bars represent the point estimate and confidence intervals of genetic correlation with each disorder. **(B)** Pairwise correlation among autoimmune disorders. The color of each checker represents the magnitudes of correlation. A darker color represents a stronger correlation. **Bonferroni correct significance, *suggestive significance. IBD, inflammatory bowel disease; UC, ulcerative colitis; CD, Crohn’s disease; RA, rheumatoid arthritis; SLE, systemic lupus erythematosus; MS, multiple sclerosis.

As shown in Figure 1A, using full-set GWAS summary data, we observed negligible shared genetic similarities of alcohol consumption with each disorder. Indeed, the genetic correlation estimates were all negative ranging from −0.07 to −0.02, meaning that the genetic variant associated with an increase in dose of alcohol tends to be associated with a decreased risk of autoimmune disorder. However, all these genetic correlations were not significant with confidence intervals including 1 and *P*-values > 0.05, contrasted by the significant pairwise genetic correlation observed among autoimmune disorders (Figure 1B).

Genetic correlation describes the intrinsic genome-wide average sharing of genetic effects between traits that are independent of environmental factors. We next performed MR analysis to elucidate a potential directional or causal association between alcohol and autoimmune disorders. We were able to match almost all alcohol-associated genetic instruments to our outcome data, ranging from 98 (99%) in IBD, 93 in RA and MS (94%), and 82 in SLE (83%)—a virtually complete coverage (Supplementary Table 4). These 99 alcohol-associated genetic variants constructed a strong IV with an overall F-statistic of 122.4.

As shown in Table 1, for most autoimmune disorders examined by us, genetically predicted alcohol consumption was associated with a slightly (10–25%) decreased risk of disease onset (IBD: OR_*IVW*_ = 0.84; UC: OR_*IVW*_ = 0.93; CD: OR_*IVW*_ = 0.70; RA: OR_*IVW*_ = 0.80; MS: OR_*IVW*_ = 0.75); for SLE, an OR_*IVW*_ of 1.10 was observed. However, all these associations were not statistically significant with confidence intervals covering 1.00 (95%CI, IBD: 0.54–1.29; UC: 0.59–1.49; CD: 0.38–1.27; RA: 0.54–1.19; MS: 0.49–1.12; SLE: 0.51–2.37) and *P*-values larger than 0.05. Such null findings were supported by the maximum likelihood method and the weighted median approach where we observed non-significant effects (although in opposite directions for RA and MS) with confidence intervals covering 1. MR–Egger regression did not reveal apparent signs of horizontal pleiotropy (*P*-values for the MR–Egger intercept, IBD: *P* = 0.64; UC: *P* = 0.90; CD: *P* = 0.40; RA: *P* = 0.09; MS: *P* = 0.26; SLE: *P* = 0.48).

**TABLE 1:** The association between genetically predicted levels of alcohol consumption and risk of common autoimmune inflammatory diseases.

**Methods**	**# SNP**	**OR (95%CI)**	***P*-value**	***P*-value for intercept**	**# SNP**	**OR (95%CI)**	***P*-value**	***P*-value for intercept**
		
	**Full-set**				**Remove palindromic SNPs**			
**Inflammatory bowel disease**								
IVW	98	0.84 (0.54–1.29)	0.42		84	0.84 (0.52–1.34)	0.46	
Maximum likelihood	98	0.84 (0.63–1.11)	0.23		84	0.84 (0.62–1.13)	0.25	
Weighted median	98	0.92 (0.56–1.51)	0.73		84	0.92 (0.56–1.52)	0.75	
MR–Egger	98	0.96 (0.46–2.00)	0.92	0.64	84	1.01 (0.47–2.21)	0.97	0.55
**Ulcerative colitis**								
IVW	98	0.93 (0.59–1.49)	0.77		84	0.92 (0.56–1.52)	0.75	
Maximum likelihood	98	0.93 (0.65–1.33)	0.70		84	0.92 (0.63–1.34)	0.66	
Weighted median	98	0.99 (0.51–1.91)	0.97		84	1.00 (0.52–1.92)	1.00	
MR–Egger	98	0.97 (0.44–2.18)	0.95	0.90	84	0.99 (0.43–2.28)	0.98	0.84
**Crohn’s disease**								
IVW	98	0.70 (0.38–1.27)	0.24		84	0.70 (0.36–1.36)	0.30	
Maximum likelihood	98	0.71 (0.48–1.03)	0.07		84	0.70 (0.47–1.03)	0.07	
Weighted median	98	0.98 (0.51–1.87)	0.95		84	0.99 (0.52–1.88)	0.98	
MR–Egger	98	0.98 (0.36–2.63)	0.97	0.40	84	1.02 (0.36–2.96)	0.96	0.37
**Rheumatoid arthritis**								
IVW	93	0.80 (0.54–1.19)	0.27		80	0.85 (0.56–1.29)	0.45	
Maximum likelihood	93	0.80 (0.56–1.14)	0.22		80	0.85 (0.58–1.24)	0.40	
Weighted median	93	1.38 (0.77–2.50)	0.28		80	1.43 (0.74–2.75)	0.29	
MR–Egger	93	1.45 (0.66–3.18)	0.36	0.09	80	1.58 (0.71–3.54)	0.27	0.08
**Multiple sclerosis**								
IVW	93	0.75 (0.49–1.12)	0.16		80	0.74 (0.47–1.16)	0.18	
Maximum likelihood	93	0.74 (0.53–1.03)	0.07		80	0.73 (0.52–1.04)	0.08	
Weighted median	93	1.13 (0.66–1.95)	0.65		80	1.13 (0.63–2.02)	0.68	
MR–Egger	93	1.17 (0.49–2.83)	0.72	0.26	80	1.27 (0.49–3.28)	0.62	0.20
**Systemic lupus erythematosus**								
IVW	82	1.10 (0.51–2.37)	0.80		70	1.14 (0.49–2.66)	0.76	
Maximum likelihood	82	1.11 (0.62–1.97)	0.73		70	1.14 (0.61–2.14)	0.67	
Weighted median	82	1.91 (0.71–5.12)	0.20		70	1.85 (0.65–5.27)	0.25	
MR–Egger	82	2.14 (0.29–15.69)	0.46	0.48	70	1.24 (0.13–11.89)	0.85	0.94

Palindromic SNPs introduce ambiguity for the identity of effect alleles in exposure and outcome data. Sensitivity analysis removing palindromic SNPs (Table 1) revealed similar null associations for all autoimmune disorders.

A search of GWAS catalog^1^ reveals considerable potential for pleiotropic effects, as some IVs were identified to be associated with important potential confounders with genome-wide significance (Supplementary Table 1). We next performed a sensitivity analysis excluding those SNPs. As shown in Table 2, consistent with our primary analysis, we did not observe any significantly altered risk of autoimmune disorders with genetic predisposition to alcohol consumption. A significantly reduced risk of RA was identified [IVW, OR (95%CI) = 0.51 (0.30–0.88)], yet such an association did not pass multiple corrections and did not remain directionally consistent in other methods [MR–Egger, OR (95%CI) = 2.08 (0.42–10.30)]. In both sensitivity analyses, no apparent horizontal pleiotropy was observed as reflected by the intercepts of MR–Egger regression (Tables 1, 2).

**TABLE 2:** Genetically predicted levels of alcohol consumption and the risk of autoimmune inflammatory diseases.

**Methods**	**# SNP**	**OR (95%CI)**	***P*-value**	***P*-value for intercept**
**Inflammatory bowel disease**				
IVW	71	0.78 (0.44–1.36)	0.38	
Maximum likelihood	71	0.77 (0.51–1.16)	0.21	
Weighted median	71	0.63 (0.33–1.21)	0.17	
MR–Egger	71	2.18 (0.50–9.59)	0.31	0.14
**Ulcerative colitis**				
IVW	71	0.89 (0.47–1.69)	0.72	
Maximum likelihood	71	0.89 (0.53–1.48)	0.64	
Weighted median	71	0.66 (0.30–1.46)	0.31	
MR–Egger	71	1.52 (0.27–8.39)	0.64	0.51
**Crohn’s disease**				
IVW	71	0.61 (0.28–1.34)	0.22	
Maximum likelihood	71	0.60 (0.34–1.04)	0.07	
Weighted median	71	1.19 (0.48–2.93)	0.71	
MR–Egger	71	3.24 (0.41–25.51)	0.27	0.09
**Rheumatoid arthritis**				
IVW	68	0.51 (0.30–0.88)	**0.02**	
Maximum likelihood	68	0.50 (0.31–0.81)	**0.005**	
Weighted median	68	0.88 (0.42–1.85)	0.73	
MR–Egger	68	2.08 (0.42–10.30)	0.37	0.07
**Multiple sclerosis**				
IVW	67	0.85 (0.50–1.42)	0.54	
Maximum likelihood	67	0.85 (0.55–1.30)	0.44	
Weighted median	67	1.12 (0.60–2.13)	0.71	
MR–Egger	67	3.01 (0.62–14.71)	0.18	0.10
**Systemic lupus erythematosus**				
IVW	60	1.24 (0.49–3.15)	0.66	
Maximum likelihood	60	1.25 (0.61–2.54)	0.54	
Weighted median	60	0.63 (0.20–2.02)	0.44	
MR–Egger	60	4.23 (0.31–57.18)	0.28	0.32

*A sensitivity analysis excluding SNPs associated with potential confounding traits.*

Inflammatory bowel disease, RA, and MS are Th1-related autoimmune disorders, meta-analyzing across these three traits yielded to a reduced effect with marginal significance [OR_*meta*_(95%CI) = 0.79 (0.63–1.01), *P* = 0.06 using all IVs; OR_*meta*_(95%CI) = 0.70 (0.51–0.95), *P* = 0.02 using IVs excluding confounders]. Meta-analyzing all four traits did not reveal any significant effect [OR_*meta*_(95%CI) = 0.82 (0.65–1.03), *P* = 0.08 using all IVs; OR_*meta*_(95%CI) = 0.74 (0.54–1.02), *P* = 0.06 using curated IVs without pleiotropic effects].

Obesity and smoking are two important environmental risk factors clustering together with alcohol intake. We therefore employed a multivariable MR approach to adjust for potential horizontal pleiotropy acting in particular through BMI and smoking. As shown in Table 3 and consistent with our sensitivity analysis, we did not observe apparent significant effects of alcohol consumption with risk of autoimmune disease after adjusting for BMI and smoking, except a suggestive reduced effect with MS which did not withstand multiple corrections (OR = 0.49 and *P* = 0.02). Leave-one-out analysis did not identify any outlying variants (Supplementary Table 5).

**TABLE 3:** Genetically predicted levels of alcohol consumption and risk of common autoimmune diseases.

**Methods**	**# SNP**	**OR (95%CI)**	***P*-value**
**Inflammatory bowel disease**			
Body mass index	48	0.94 (0.42–2.11)	0.89
Smoking status	97	0.80 (0.51–1.30)	0.38
**Ulcerative colitis**			
Body mass index	48	1.20 (0.52–2.75)	0.67
Smoking status	97	0.94 (0.56–1.59)	0.83
**Crohn’s disease**			
Body mass index	48	0.56 (0.17–1.82)	0.34
Smoking status	97	0.64 (0.33–1.25)	0.19
**Rheumatoid arthritis**			
Body mass index	48	0.66 (0.36–1.21)	0.18
Smoking status	92	0.79 (0.50–1.24)	0.31
**Multiple sclerosis**			
Body mass index	48	0.49 (0.26–0.91)	**0.02**
Smoking status	92	0.79 (0.49–1.27)	0.33
**Systemic lupus erythematosus**			
Body mass index	42	0.86 (0.31–2.38)	0.77
Smoking status	93	0.83 (0.34–2.03)	0.69

*Multivariable analysis adjusting for the effect of body mass index and smoking status.*

**FIGURE 2:**
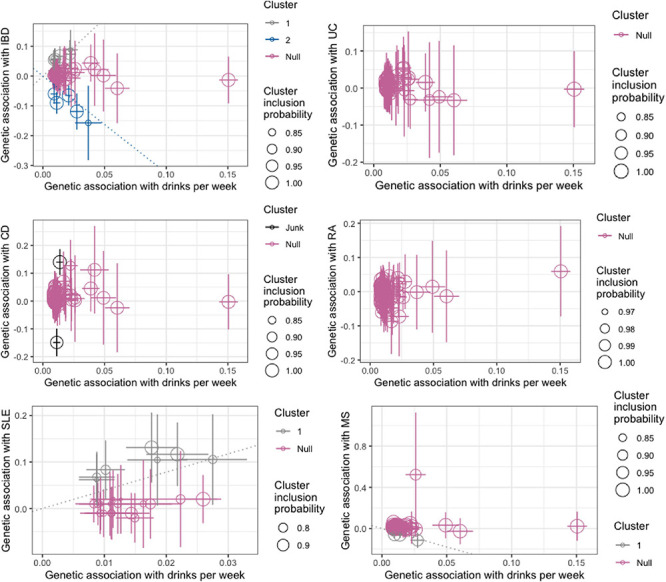
Genetic associations with alcohol consumption (drinks per week) and risk of autoimmune inflammatory diseases (log odds) per additional alcohol consumption increasing alleles. Each genetic variant is represented by a point. Error bars are 95% confidence intervals for the genetic associations. Colors represent the clusters. Variants are only assigned to the cluster if the conditional probability is >0.8 and cluster only displayed if at least four variants are assigned to the cluster. IBD, inflammatory bowel disease; UC, ulcerative colitis; CD, Crohn’s disease; RA, rheumatoid arthritis; SLE, systemic lupus erythematosus; MS, multiple sclerosis.

Alcohol consumption-associated variants may influence the risk of autoimmune diseases via distinct biological mechanisms. We therefore examined a scenario where variants can be divided into different clusters. According to MR-Clust, each IV is only assigned to a cluster if the conditional probability of belonging to that cluster is high (larger than 0.8) and clusters are only displayed if at least four IVs are assigned to it (Foley et al., 2019). As shown in Figure 2, for IBD, we observed two distinct clusters suggesting one strong positive causal effect and one strong negative causal effect; for SLE, we observed a single cluster suggesting a strong positive causal effect; and for MS, we observed a single cluster suggesting a strong negative causal effect. However, when we performed MR-Clust analysis excluding confounding IVs (corresponding to IVs used in Table 2), all previously observed clusters disappeared, largely consistent with an overall null finding (data not shown).

Finally, we complemented our main results by incorporating IVs associated with excessive or harmful drinking behavior (AUDIT, *N* = 13). As shown in Table 4 and consistent with our main findings, excessive drinking did not appear to reduce the risk of autoimmune disorders. On the contrary, we observed an increased non-significant risk of IBD (OR = 1.21; 1.25 for UC and 1.14 for CD) and RA (OR = 1.16) with harmful drinking. We stress caution when interpreting these results given the very few genetic instruments associated with AUDIT.

**TABLE 4:** The association between genetically predicted levels of harmful alcohol consumption (alcohol use disorder identification test score) and risk of common autoimmune inflammatory diseases.

**Methods**	**# SNP**	**OR (95%CI)**	***P*-value**	***P*-value for intercept**	**# SNP**	**OR (95%CI)**	***P*-value**	***P*-value for intercept**
		
	**Full-set**				**Remove SNPs associated with confounders**			
**Inflammatory bowel disease**								
IVW	13	0.86 (0.63–1.16)	0.33		9	1.21 (0.87–1.70)	0.26	
MR–Egger	13	0.83 (0.52–1.31)	0.44	0.83	9	0.62 (0.15–2.51)	0.52	0.36
**Ulcerative colitis**								
IVW	13	0.95 (0.73–1.24)	0.70		9	1.25 (0.86–1.81)	0.24	
MR–Egger	13	0.90 (0.60–1.36)	0.64	0.76	9	0.40 (0.08–2.15)	0.32	0.21
**Crohn’s disease**								
IVW	13	0.77 (0.50–1.21)	0.26		9	1.14 (0.72–1.78)	0.58	
MR–Egger	13	0.80 (0.41–1.56)	0.52	0.89	9	0.90 (0.12–6.51)	0.92	0.82
**Rheumatoid arthritis**								
IVW	13	**1.20 (1.00–1.44)**	**0.05**		9	1.16 (0.82–1.64)	0.42	
MR–Egger	13	1.23 (0.82–1.83)	0.34	0.91	9	1.15 (0.22–5.99)	0.87	1.00
**Multiple sclerosis**								
IVW	12	0.91 (0.70–1.18)	0.46		8	0.95 (0.69–1.32)	0.76	
MR–Egger	12	0.85 (0.51–1.39)	0.53	0.76	8	0.78 (0.20–3.04)	0.74	0.78
**Systemic lupus erythematosus**								
IVW	10	1.04 (0.64–1.69)	0.86		8	0.95 (0.59–1.54)	0.85	
MR–Egger	10	1.60 (0.40–6.37)	0.53	0.54	8	0.50 (0.10–2.53)	0.43	0.44

## Discussion

We conducted a large-scale comprehensive genetic analysis to systemically interrogate the role of alcohol consumption in several common autoimmune inflammatory disorders. Overall, alcohol consumption and autoimmune disorder share a reverse yet non-significant genetic basis. Despite a few suggestive significant findings from MR in support of alcohol intake and a reduced risk of RA and MS, these results did not withstand multiple corrections. Meta-analyzing all traits did not reveal significant effects, and meta-analyzing three Th1-related disorders (IBD, RA, and MS) yielded to a reduced effect with significance (*P* = 0.02) not withstanding multiple corrections. Therefore, we consider an overall null association as our main conclusion.

To the best of our knowledge, the current MR study is the largest in sample size of its kind, leveraging information on 99 genetic instruments and involving data from more than one million individuals of European ancestry (941,280 individuals for exposure and 157,723 individuals for outcome). Two MR studies have been conducted for alcohol use and autoimmune disorder; none had the opportunity to achieve our power. For example, Bae and Bae and Lee (2019a, b) examined the causal relationship of alcohol intake with risk of RA and SLE, using approximately 20 alcohol-associated genome-wide significant SNPs as IVs. For outcomes, two meta-GWAS(s) were included, one with 5,539 autoantibody-positive RA patients (and 20,169 controls) and the other with 1,311 lupus patients (and 1,783 controls). No evidence of a causal relationship was identified for either RA [OR (95%CI) = 1.24 (0.82–1.89), *P* = 0.31] or lupus [OR (95%CI) = 0.46 (0.07–2.94), *P* = 0.42]. It is very likely that the few IVs did not fully capture the effect of alcohol. Our current study, with a largely augmented sample size and by incorporating additional alcohol consumption associated loci, greatly improved the strength of genetic instruments (F-statistic = 122.4) as well as both the accuracy and precision of MR estimates, as compared with previous findings.

We found an overall protective effect of alcohol intake on the three Th1-mediated autoimmune disorders (IBD, RA, and MS) as a whole; however, when breaking down into individual disorders, we did not find convincing evidence in support of a beneficial role of alcohol consumption. Our conclusion, although consistent with previous small-scale MR studies, is not supported by observational studies. For example, Jin et al. (2014) summarized results from eight prospective studies containing 195,029 participants and 1,878 RA cases and found that low to moderate alcohol consumption yielded a preventive effect on the disease development [RR (95%CI) = 0.86 (0.78–0.94)]. Moreover, Wang et al. (2008) conducted a meta-analysis including six case–control studies and one cohort study and found a significantly decreased risk of lupus with moderate alcohol drinking [OR (95%CI) = 0.72 (0.55–0.95)]. Further, Zhu et al. (2015) aggregated data from nine case–control studies and one cohort study and identified an OR for the association between alcohol consumption and MS to be 0.91 (95%CI = 0.39–2.41). Reasons underlying such discrepancies can be multifactorial. Results from observational studies are likely to be plagued by measurement error or biases. For example, assessment of alcohol consumption is usually done by questionnaires, where frequency and amount of consumption are collected—precisely determining the amount of consumed alcohol is difficult. Indeed, alcohol intake can be expressed as a single measurement with “low,” “medium,” and “high” categories; such categorical measurement may however be of limited resolution.

Our study has several advantages in addition to its large sample size. We restricted participants to individuals of European ancestry which largely controlled for bias arising from population stratification as compared to using mixed ethnicity populations. We interrogated four common autoimmune disorders which greatly expanded pervious findings. We conducted several important sensitivity analyses to verify MR model assumptions. We selected the most significant independent SNPs identified by the largest alcohol GWAS, so all were robustly and strongly associated with exposure of interest, guaranteeing “relevance” assumption. We excluded SNPs associated with potential confounders on the exposure–outcome relationship as confirmed by GWAS catalog, to satisfy “exclusion restriction” assumption.

Nevertheless, insufficient power remains a common limitation of MR studies, because genetic variants usually explain a modest proportion of phenotypic variance. This is also a concern for alcohol consumption, a complex human behavior largely influenced by non-genetic factors. Our non-significant findings are perhaps not surprising, considering that the 99 currently reported alcohol-associated SNPs only explain ∼1% of phenotypic variance. Although improvement in the proportion of variability explained by IVs was modest, our overall statistical power was considerably raised using data from substantially augmented GWASs of four autoimmune disorders. We had 80% power at an alpha level of 0.05 to identify a ∼25–30% relative decreased risk of IBD, RA, MS, and lupus (i.e., an OR of 0.70–0.75) per SD increase in alcohol consumption. We note that most of our estimated ORs are in the expected direction, and the suggested associations for the three Th-1-mediated autoimmune diseases are in line with what have been observed previously in studies based on self-reported alcohol consumption.

Alcohol consumption plays a complicated role in human health as its effect on diseases depends on dose. In most autoimmune diseases, moderate weekly intake shows the lowest disease incidence. Such a U-shaped or J-shaped relationship cannot be identified by MR design with only summary-level data which is set out for a linear relationship. It has been proposed that a high dose of alcohol can directly suppress a wide range of immune responses (Romeo et al., 2007). We try to address this question by incorporating IVs associated with harmful drinking behavior; yet, excessive drinking does not appear to reduce the risk of autoimmune disorders. We stress caution when interpreting these results given the very few instruments available for AUDIT. Another major hypothesis for the null association is the heterogeneity of phenotypes. For example, RA can be divided into different subsets based on seropositivity. This means that even though the association is null with overall disease, signals may appear when we subtype the outcome. It is also likely that alcohol consumption, albeit with no convincing evidence to demonstrate a causal link with disease risk, may complicate symptoms or aggravate disease prognosis.

To conclude, our updated analysis, with its greatly augmented sample size and substantially improved statistical power, does not convincingly support a beneficial role of alcohol consumption in autoimmune disorder. Our findings should be interpreted with caution. Future studies may be performed to update our findings when additional alcohol-associated IVs are revealed by GWAS analysis; as well as to explore a non-linear relationship (capitalizing on individual-level data) or to understand the impact on disease prognosis.

## Data Availability Statement

The original contributions presented in the study are included in the article/Supplementary Material, further inquiries can be directed to the corresponding author.

## Author Contributions

XJ, ZZ, and AM analyzed and interpreted the data regarding genetic correlation and mendelian randomization. LA, IK, and TO contributed significantly in writing and modifying the manuscript. All authors read and approved the final manuscript.

## Conflict of Interest

The authors declare that the research was conducted in the absence of any commercial or financial relationships that could be construed as a potential conflict of interest.

## Footnotes

1 https://www.ebi.ac.uk/gwas/
