# Supplementary material for: Alcohol Consumption and Risk of Common Autoimmune Inflammatory Diseases—Evidence From a Large-Scale Genetic Analysis Totaling 1 Million Individuals
Source: Front Genet. 2021 Jun 22;12:687745. doi: 10.3389/fgene.2021.687745 (PMC8258244; doi:10.3389/fgene.2021.687745)
Supplement: Supplementary file 1 [file Table_1.docx]

| **Supplementary Table 1. The characteristic of alcohol consumption associated index SNPs, their effect sizes and associations with potential confounders.** | | | | | | | | |
| --- | --- | --- | --- | --- | --- | --- | --- | --- |
| **SNP** | **Chr** | **Position** | **Allele frequency** | **A1** | **A2** | **Exposure** | | **Confounder** |
|  |  |  |  |  |  | **beta** | **se** |  |
| rs705687 | 1 | 4548453 | 0,79 | G | A | -0,011 | 0,002 |  |
| rs58107686 | 1 | 33837334 | 0,33 | A | C | -0,010 | 0,002 |  |
| rs12088813 | 1 | 66407700 | 0,27 | C | A | -0,009 | 0,002 |  |
| rs5024204 | 1 | 71491890 | 0,28 | T | A | 0,010 | 0,002 |  |
| rs10753661 | 1 | 165119792 | 0,68 | A | G | -0,009 | 0,002 |  |
| rs28680958 | 1 | 173848808 | 0,22 | A | G | -0,011 | 0,002 | Well-being spectrum, neuroticism, depressive symptoms |
| rs823114 | 1 | 205719532 | 0,55 | A | G | 0,009 | 0,001 | Body mass index, Pakinson's disease |
| rs77165542 | 2 | 430975 | 0,03 | T | C | -0,026 | 0,004 | Anxiety/tension (special factor of neuroticism) |
| rs1260326 | 2 | 27730940 | 0,60 | C | T | 0,021 | 0,001 | Platelet count, T2D, gout, metabolite level, body mass index, lipid level, height, Crohn's disease |
| rs2178197 | 2 | 27860551 | 0,57 | G | A | -0,009 | 0,001 |  |
| rs13383034 | 2 | 45155276 | 0,33 | T | C | 0,015 | 0,002 |  |
| rs1004787 | 2 | 45159091 | 0,55 | A | G | 0,008 | 0,001 | Smoking status |
| rs13032049 | 2 | 63581507 | 0,28 | G | A | 0,010 | 0,002 |  |
| rs828867 | 2 | 74334462 | 0,55 | A | G | 0,009 | 0,001 | Educational attainment, smoking |
| rs11692435 | 2 | 98275354 | 0,09 | A | G | 0,017 | 0,003 | Smoking initiation, body mass index, colorectal cancer |
| rs13024996 | 2 | 144225215 | 0,36 | A | C | -0,011 | 0,002 |  |
| rs72859280 | 2 | 147956293 | 0,04 | T | G | 0,023 | 0,004 |  |
| rs56337305 | 2 | 225475560 | 0,38 | C | T | -0,010 | 0,001 |  |
| rs13094887 | 3 | 70968431 | 0,30 | T | A | -0,010 | 0,002 |  |
| rs62250685 | 3 | 85457240 | 0,61 | G | A | -0,014 | 0,002 |  |
| rs74664784 | 3 | 85475292 | 0,36 | C | T | -0,013 | 0,002 | Smoking initiation (ever regular vs never regular) |
| rs13066454 | 3 | 93994255 | 0,40 | T | C | -0,009 | 0,001 |  |
| rs9838144 | 3 | 131576287 | 0,21 | C | G | -0,010 | 0,002 |  |
| rs2011092 | 3 | 141124607 | 0,34 | C | T | -0,009 | 0,002 |  |
| rs60654199 | 3 | 141267295 | 0,06 | A | C | -0,017 | 0,003 |  |
| rs6787172 | 3 | 158187811 | 0,55 | G | T | -0,008 | 0,001 | Subjective well-being |
| rs3748034 | 4 | 3446091 | 0,14 | T | G | -0,012 | 0,002 | Hepatocyte growth factor levels |
| rs7682824 | 4 | 39406254 | 0,55 | T | C | 0,008 | 0,002 |  |
| rs11940694 | 4 | 39414993 | 0,60 | G | A | 0,026 | 0,001 | Blood urea nitrogen levels |
| rs35538052 | 4 | 39418965 | 0,38 | A | G | -0,009 | 0,002 |  |
| rs4501255 | 4 | 42151306 | 0,24 | G | C | 0,011 | 0,002 |  |
| rs12499107 | 4 | 99678691 | 0,13 | G | A | 0,013 | 0,002 |  |
| rs144198753 | 4 | 99713350 | 0,02 | T | C | -0,042 | 0,006 |  |
| rs1154414 | 4 | 100000136 | 0,14 | C | T | 0,018 | 0,002 |  |
| rs1229984 | 4 | 100239319 | 0,96 | C | T | 0,151 | 0,004 | Body mass index, cadiovascular disease, esophageal cancer, oral cavity cancer |
| rs10028756 | 4 | 100254520 | 0,13 | A | G | -0,019 | 0,002 |  |
| rs561222871 | 4 | 100260679 | 0,05 | T | C | -0,039 | 0,004 |  |
| rs36052336 | 4 | 100273594 | 0,06 | G | A | -0,018 | 0,003 |  |
| rs2165670 | 4 | 100286085 | 0,11 | A | G | 0,023 | 0,002 |  |
| rs17029090 | 4 | 100443853 | 0,02 | G | A | -0,049 | 0,005 |  |
| rs79139602 | 4 | 100444363 | 0,02 | T | A | 0,060 | 0,005 |  |
| rs4699791 | 4 | 101243023 | 0,10 | A | G | 0,019 | 0,002 |  |
| rs13107325 | 4 | 103188709 | 0,07 | T | C | -0,028 | 0,003 | Schizophrenia, body mass index, educational attainment, cognitive performance, HDL, waist-hip ratio |
| rs4690727 | 4 | 143648579 | 0,72 | G | C | 0,011 | 0,002 |  |
| rs10004020 | 4 | 152968372 | 0,72 | A | G | 0,009 | 0,002 |  |
| rs12651313 | 4 | 171086393 | 0,44 | G | C | -0,009 | 0,001 |  |
| rs4916723 | 5 | 87854395 | 0,42 | C | A | -0,010 | 0,001 | Neuroticism, autism, ADHD |
| rs12655091 | 5 | 144412335 | 0,53 | A | G | -0,008 | 0,001 |  |
| rs55872084 | 5 | 155902003 | 0,24 | T | G | 0,010 | 0,002 |  |
| rs11739827 | 5 | 166803321 | 0,45 | T | G | -0,008 | 0,001 |  |
| rs10085696 | 7 | 69783020 | 0,19 | G | A | -0,011 | 0,002 |  |
| rs6460047 | 7 | 73042443 | 0,21 | C | T | 0,012 | 0,002 | Serum uric acid levels |
| rs10236149 | 7 | 98977515 | 0,12 | G | A | -0,013 | 0,002 |  |
| rs35034355 | 7 | 103840115 | 0,52 | A | G | -0,008 | 0,001 |  |
| rs6951574 | 7 | 153489744 | 0,46 | C | T | 0,013 | 0,001 | Smoking status |
| rs13250583 | 8 | 20949917 | 0,21 | T | C | -0,010 | 0,002 |  |
| rs1217091 | 8 | 64527399 | 0,81 | C | T | 0,012 | 0,002 |  |
| rs28601761 | 8 | 126500031 | 0,42 | G | C | 0,009 | 0,001 | Cardiovascular disease |
| rs55932213 | 9 | 108755622 | 0,74 | G | A | 0,009 | 0,002 |  |
| rs10978550 | 9 | 109345993 | 0,21 | C | T | -0,012 | 0,002 |  |
| rs7074871 | 10 | 110507806 | 0,26 | A | G | -0,009 | 0,002 |  |
| rs17665139 | 10 | 125093880 | 0,15 | T | C | -0,012 | 0,002 |  |
| rs7950166 | 11 | 8642218 | 0,64 | T | C | -0,010 | 0,002 |  |
| rs11030084 | 11 | 27643725 | 0,18 | T | C | -0,011 | 0,002 | Educational attainment (years of education) |
| rs56030824 | 11 | 47397353 | 0,32 | A | G | -0,012 | 0,002 |  |
| rs10750025 | 11 | 113424042 | 0,69 | T | C | 0,010 | 0,002 |  |
| rs1713676 | 11 | 113660576 | 0,52 | G | A | -0,008 | 0,001 | Smoking initiation |
| rs4938230 | 11 | 116075001 | 0,84 | A | C | 0,013 | 0,002 |  |
| rs682011 | 11 | 121544285 | 0,56 | C | T | 0,008 | 0,001 |  |
| rs12795042 | 11 | 133658168 | 0,62 | C | A | -0,008 | 0,002 |  |
| rs10876188 | 12 | 51895882 | 0,46 | T | C | -0,008 | 0,001 |  |
| rs3809162 | 12 | 54674235 | 0,40 | G | A | 0,009 | 0,001 |  |
| rs10506274 | 12 | 81601464 | 0,48 | T | G | -0,009 | 0,001 |  |
| rs4842786 | 12 | 92170791 | 0,58 | A | G | -0,009 | 0,001 |  |
| rs500321 | 13 | 27124360 | 0,74 | T | A | -0,010 | 0,002 |  |
| rs1123285 | 14 | 57274519 | 0,34 | G | C | -0,009 | 0,002 | Educational attainment (years of education) |
| rs2180870 | 14 | 58782779 | 0,14 | C | T | -0,012 | 0,002 |  |
| rs28929474 | 14 | 94844947 | 0,02 | T | C | -0,037 | 0,005 | Height, blood pressure, metabolite levels, fat-free mass, lung function |
| rs11625650 | 14 | 104610138 | 0,23 | A | G | -0,010 | 0,002 |  |
| rs2472297 | 15 | 75027880 | 0,25 | T | C | 0,011 | 0,002 | Coffee consumption |
| rs12907323 | 15 | 86796012 | 0,41 | G | A | 0,008 | 0,001 |  |
| rs2764771 | 16 | 20013793 | 0,31 | A | G | 0,010 | 0,002 |  |
| rs17177078 | 16 | 24810681 | 0,06 | T | C | -0,022 | 0,003 | Type 2 diabetes (dietary heme iron intake interaction) |
| rs378421 | 16 | 28754684 | 0,40 | A | G | -0,011 | 0,001 |  |
| rs113443718 | 16 | 29892184 | 0,31 | A | G | -0,010 | 0,002 |  |
| rs62044525 | 16 | 64872590 | 0,18 | G | C | -0,012 | 0,002 |  |
| rs7185555 | 16 | 69131281 | 0,15 | C | G | -0,011 | 0,002 | Diastolic blood pressure |
| rs79616692 | 16 | 72338507 | 0,11 | C | G | 0,016 | 0,002 |  |
| rs1104608 | 16 | 73912588 | 0,43 | C | G | -0,011 | 0,001 |  |
| rs4548913 | 17 | 2209888 | 0,63 | A | G | -0,008 | 0,002 |  |
| rs3803800 | 17 | 7462969 | 0,79 | G | A | 0,011 | 0,002 | IgA nephropathy, IgA levels, albumin-globulin ratio |
| rs2854334 | 17 | 29715500 | 0,62 | G | A | 0,009 | 0,001 |  |
| rs2532276 | 17 | 44246624 | 0,22 | A | C | -0,022 | 0,003 |  |
| rs10438820 | 17 | 78524597 | 0,70 | T | C | 0,009 | 0,002 |  |
| rs9950000 | 18 | 53052169 | 0,40 | T | C | -0,009 | 0,001 |  |
| rs4092465 | 18 | 55080437 | 0,64 | G | A | -0,008 | 0,002 | C-reactive protein levels |
| rs281379 | 19 | 49214274 | 0,51 | A | G | 0,014 | 0,001 | Crohn's disease |
| rs4815364 | 20 | 25035711 | 0,62 | A | G | 0,009 | 0,001 |  |
| rs9607814 | 22 | 41946519 | 0,20 | A | C | -0,010 | 0,002 |  |
| A1: effect allele; A2: other allele. | | | | | | | | |

| **Supplementary Table 2. The characteristic of index SNPs associated with excessive drinking (AUDIT), their effect sizes with exposure and their associations with potential confounders.** | | | | | | | | |
| --- | --- | --- | --- | --- | --- | --- | --- | --- |
| **SNP** | **Chr** | **Position** | **Allele frequency** | **A1** | **A2** | **Exposure** | | **Confounder** |
|  |  |  |  |  |  | **beta** | **se** |  |
| rs1260326 | 2 | 27730940 | 0,591 | C | T | 0,045 | 0,0054 | Triglycerides, lipids |
| rs2717071 | 2 | 58030962 | 0,628 | A | G | 0,034 | 0,0055 |  |
| rs12639940 | 4 | 39420981 | 0,614 | A | G | 0,033 | 0,0056 |  |
| rs1229984 | 4 | 100239319 | 0,970 | C | T | 0,340 | 0,0159 | oral cancer |
| rs1229978 | 4 | 100256199 | 0,403 | C | T | 0,042 | 0,0055 |  |
| rs13107325 | 4 | 103188709 | 0,921 | C | T | 0,103 | 0,0099 | LDL, HDL, BMI, blood pressure |
| rs62339861 | 4 | 150983421 | 0,792 | C | T | 0,037 | 0,0067 |  |
| rs2961817 | 5 | 50443063 | 0,657 | A | G | 0,033 | 0,0057 |  |
| rs185177474 | 5 | 143889333 | 0,031 | A | C | 0,089 | 0,0158 |  |
| rs12425096 | 12 | 51980459 | 0,775 | A | C | 0,035 | 0,0064 |  |
| rs9937709 | 16 | 53820813 | 0,587 | A | G | 0,044 | 0,0054 |  |
| rs4794018 | 17 | 47093398 | 0,337 | C | T | 0,032 | 0,0058 |  |
| rs35572189 | 17 | 79419025 | 0,368 | A | G | 0,035 | 0,0056 | Urinary albumin excretion |

| **Supplementary Table 3. Four common autoimmune inflammatory disorders.** | | | | | | | |
| --- | --- | --- | --- | --- | --- | --- | --- |
| **Outcome trait** | **Author** | **Year** | **Journal** | **PMID** | **Population** | **Cases** | **Controls** |
| Inflammatory bowel disease | Liu JZ | 2015 | Nat Genet | 26192919 | European | 12 882 | 21 770 |
| Ulcerative colitis | Liu JZ | 2015 | Nat Genet | 26192919 | European | 6 968 | 20 464 |
| Crohn's disease | Liu JZ | 2015 | Nat Genet | 26192919 | European | 5 956 | 14 927 |
| Rheumatoid arthritis | Okada Y | 2014 | Nature | 24390342 | European | 14 361 | 43 923 |
| Multiple sclerosis | IMSGC | 2019 | Science | 31604244 | European | 14 802 | 26 703 |
| Systemic lupus erythematosus | Bentham J | 2015 | Nat Genet | 26502338 | European | 7 291 | 15 991 |

| **Supplementary Table 4. The effect sizes of alcohol consumption associated index SNPs with disease outcomes.** | | | | | | | | | | | | | | | | | |
| --- | --- | --- | --- | --- | --- | --- | --- | --- | --- | --- | --- | --- | --- | --- | --- | --- | --- |
| **SNP** | **A1** | **A2** | **beta exposure** | **se exposure** | **beta IBD** | **se IBD** | **beta UC** | **se UC** | **beta CD** | **se CD** | **beta RA** | **se RA** | **beta MS** | **se MS** | **beta SLE** | **se SLE** |  |
| rs10004020 | A | G | 0,009 | 0,002 | -0,009 | 0,019 | 0,005 | 0,024 | -0,028 | 0,025 | 0,024 | 0,022 | -0,002 | 0,018 | NA | NA |  |
| rs10028756 | A | G | -0,019 | 0,002 | 0,015 | 0,025 | 0,044 | 0,032 | -0,033 | 0,034 | 0,013 | 0,029 | -0,034 | 0,024 | 0,122 | 0,040 |  |
| rs1004787 | A | G | 0,008 | 0,001 | -0,024 | 0,017 | -0,030 | 0,022 | -0,021 | 0,024 | 0,036 | 0,020 | -0,011 | 0,017 | -0,030 | 0,028 |  |
| rs10085696 | G | A | -0,011 | 0,002 | -0,017 | 0,021 | -0,011 | 0,027 | -0,031 | 0,029 | -0,005 | 0,025 | 0,013 | 0,021 | -0,020 | 0,032 |  |
| rs10236149 | G | A | -0,013 | 0,002 | 0,014 | 0,027 | 0,021 | 0,033 | 0,016 | 0,036 | 0,061 | 0,029 | 0,040 | 0,024 | NA | NA |  |
| rs10438820 | T | C | 0,009 | 0,002 | 0,010 | 0,018 | 0,009 | 0,023 | 0,020 | 0,025 | 0,008 | 0,021 | -0,026 | 0,018 | 0,010 | 0,023 |  |
| rs10506274 | T | G | -0,009 | 0,001 | -0,015 | 0,017 | -0,007 | 0,021 | -0,036 | 0,023 | 0,040 | 0,019 | 0,000 | 0,021 | NA | NA |  |
| rs10750025 | T | C | 0,010 | 0,002 | 0,026 | 0,019 | 0,026 | 0,024 | 0,006 | 0,025 | 0,005 | 0,017 | 0,025 | 0,018 | 0,020 | 0,030 |  |
| rs10753661 | A | G | -0,009 | 0,002 | 0,007 | 0,018 | 0,004 | 0,023 | 0,007 | 0,025 | -0,002 | 0,021 | -0,021 | 0,018 | NA | NA |  |
| rs10876188 | T | C | -0,008 | 0,001 | 0,022 | 0,017 | 0,015 | 0,021 | 0,045 | 0,023 | -0,018 | 0,019 | 0,044 | 0,016 | 0,010 | 0,039 |  |
| rs10978550 | C | T | -0,012 | 0,002 | -0,012 | 0,021 | 0,012 | 0,026 | -0,031 | 0,029 | 0,025 | 0,019 | -0,003 | 0,020 | 0,058 | 0,032 |  |
| rs11030084 | T | C | -0,011 | 0,002 | -0,039 | 0,021 | -0,068 | 0,027 | 0,000 | 0,029 | 0,014 | 0,025 | -0,007 | 0,021 | NA | NA |  |
| rs1104608 | C | G | -0,011 | 0,001 | 0,018 | 0,018 | 0,015 | 0,023 | 0,023 | 0,025 | -0,007 | 0,021 | 0,019 | 0,020 | 0,049 | 0,030 |  |
| rs1123285 | G | C | -0,009 | 0,002 | -0,036 | 0,018 | -0,044 | 0,023 | -0,018 | 0,024 | -0,020 | 0,021 | -0,007 | 0,017 | 0,049 | 0,032 |  |
| rs113443718 | A | G | -0,010 | 0,002 | 0,010 | 0,019 | 0,027 | 0,024 | 0,008 | 0,026 | 0,035 | 0,022 | 0,021 | 0,019 | -0,083 | 0,032 |  |
| rs1154414 | C | T | 0,018 | 0,002 | -0,014 | 0,024 | 0,011 | 0,030 | -0,061 | 0,033 | 0,000 | 0,030 | 0,025 | 0,027 | 0,131 | 0,038 |  |
| rs11625650 | A | G | -0,010 | 0,002 | -0,004 | 0,023 | -0,037 | 0,030 | 0,021 | 0,030 | 0,028 | 0,030 | -0,004 | 0,021 | -0,030 | 0,040 |  |
| rs11692435 | A | G | 0,017 | 0,003 | 0,036 | 0,033 | 0,034 | 0,042 | 0,040 | 0,045 | -0,048 | 0,040 | -0,010 | 0,040 | 0,010 | 0,038 |  |
| rs11739827 | T | G | -0,008 | 0,001 | 0,017 | 0,017 | 0,022 | 0,021 | 0,016 | 0,023 | -0,018 | 0,020 | -0,002 | 0,017 | -0,030 | 0,026 |  |
| rs11940694 | G | A | 0,026 | 0,001 | 0,024 | 0,017 | 0,028 | 0,022 | 0,003 | 0,024 | -0,016 | 0,020 | -0,008 | 0,017 | 0,020 | 0,026 |  |
| rs12088813 | C | A | -0,009 | 0,002 | -0,008 | 0,019 | -0,005 | 0,024 | -0,013 | 0,026 | -0,019 | 0,022 | -0,005 | 0,018 | 0,010 | 0,023 |  |
| rs1217091 | C | T | 0,012 | 0,002 | 0,015 | 0,022 | 0,007 | 0,027 | 0,018 | 0,029 | -0,020 | 0,025 | -0,019 | 0,021 | 0,062 | 0,040 |  |
| rs1229984 | C | T | 0,151 | 0,004 | -0,013 | 0,040 | -0,003 | 0,052 | -0,003 | 0,051 | 0,059 | 0,067 | 0,022 | 0,072 | NA | NA |  |
| rs12499107 | G | A | 0,013 | 0,002 | -0,044 | 0,026 | -0,041 | 0,032 | -0,042 | 0,036 | -0,039 | 0,031 | 0,034 | 0,028 | 0,020 | 0,038 |  |
| rs1260326 | C | T | 0,021 | 0,001 | -0,065 | 0,017 | -0,064 | 0,022 | -0,082 | 0,023 | 0,016 | 0,016 | 0,009 | 0,017 | -0,049 | 0,029 |  |
| rs12651313 | G | C | -0,009 | 0,001 | 0,010 | 0,017 | 0,013 | 0,021 | 0,011 | 0,023 | 0,000 | 0,019 | -0,008 | 0,016 | 0,020 | 0,019 |  |
| rs12655091 | A | G | -0,008 | 0,001 | -0,004 | 0,017 | -0,022 | 0,021 | 0,026 | 0,023 | -0,004 | 0,019 | 0,040 | 0,016 | 0,020 | 0,028 |  |
| rs12795042 | C | A | -0,008 | 0,002 | -0,010 | 0,018 | -0,011 | 0,023 | 0,005 | 0,025 | 0,029 | 0,021 | 0,008 | 0,019 | NA | NA |  |
| rs12907323 | G | A | 0,008 | 0,001 | 0,017 | 0,017 | 0,008 | 0,021 | 0,030 | 0,023 | -0,062 | 0,020 | -0,005 | 0,016 | -0,051 | 0,032 |  |
| rs13024996 | A | C | -0,011 | 0,002 | 0,034 | 0,018 | 0,017 | 0,022 | 0,033 | 0,024 | -0,015 | 0,020 | 0,008 | 0,017 | -0,010 | 0,035 |  |
| rs13032049 | G | A | 0,010 | 0,002 | -0,004 | 0,018 | -0,022 | 0,023 | 0,009 | 0,025 | -0,049 | 0,022 | -0,025 | 0,018 | -0,073 | 0,034 |  |
| rs13066454 | T | C | -0,009 | 0,001 | 0,002 | 0,017 | 0,004 | 0,022 | -0,001 | 0,024 | 0,003 | 0,020 | -0,010 | 0,017 | 0,030 | 0,031 |  |
| rs13094887 | T | A | -0,010 | 0,002 | 0,024 | 0,018 | 0,020 | 0,023 | 0,034 | 0,025 | 0,035 | 0,022 | 0,036 | 0,018 | NA | NA |  |
| rs13107325 | T | C | -0,028 | 0,003 | 0,119 | 0,031 | 0,031 | 0,040 | 0,222 | 0,040 | -0,029 | 0,041 | 0,114 | 0,038 | -0,105 | 0,050 |  |
| rs13250583 | T | C | -0,010 | 0,002 | 0,038 | 0,021 | 0,027 | 0,026 | 0,058 | 0,028 | 0,008 | 0,024 | 0,053 | 0,020 | 0,020 | 0,036 |  |
| rs13383034 | T | C | 0,015 | 0,002 | -0,036 | 0,018 | -0,030 | 0,023 | -0,069 | 0,025 | 0,046 | 0,021 | -0,002 | 0,018 | -0,020 | 0,027 |  |
| rs144198753 | T | C | -0,042 | 0,006 | -0,022 | 0,062 | 0,032 | 0,080 | -0,112 | 0,080 | NA | NA | NA | NA | -0,128 | 0,092 |  |
| rs17029090 | G | A | -0,049 | 0,005 | -0,002 | 0,062 | 0,024 | 0,077 | -0,012 | 0,086 | -0,014 | 0,069 | -0,032 | 0,065 | 0,104 | 0,102 |  |
| rs1713676 | G | A | -0,008 | 0,001 | -0,001 | 0,017 | 0,006 | 0,021 | -0,004 | 0,023 | 0,013 | 0,020 | 0,015 | 0,016 | -0,010 | 0,049 |  |
| rs17177078 | T | C | -0,022 | 0,003 | -0,088 | 0,034 | -0,054 | 0,043 | -0,127 | 0,047 | 0,014 | 0,032 | 0,001 | 0,032 | -0,020 | 0,053 |  |
| rs17665139 | T | C | -0,012 | 0,002 | 0,004 | 0,024 | -0,001 | 0,030 | -0,003 | 0,032 | 0,021 | 0,027 | -0,015 | 0,023 | -0,073 | 0,043 |  |
| rs2011092 | C | T | -0,009 | 0,002 | 0,029 | 0,018 | -0,006 | 0,022 | 0,069 | 0,024 | 0,023 | 0,016 | -0,062 | 0,017 | -0,020 | 0,027 |  |
| rs2165670 | A | G | 0,023 | 0,002 | 0,024 | 0,029 | 0,029 | 0,036 | 0,005 | 0,040 | 0,023 | 0,032 | 0,021 | 0,027 | -0,073 | 0,047 |  |
| rs2178197 | G | A | -0,009 | 0,001 | -0,022 | 0,017 | -0,058 | 0,021 | 0,020 | 0,023 | 0,016 | 0,020 | 0,022 | 0,016 | -0,068 | 0,028 |  |
| rs2180870 | C | T | -0,012 | 0,002 | 0,041 | 0,025 | 0,010 | 0,031 | 0,078 | 0,033 | 0,057 | 0,029 | 0,022 | 0,024 | 0,039 | 0,041 |  |
| rs2472297 | T | C | 0,011 | 0,002 | 0,015 | 0,020 | 0,030 | 0,025 | -0,016 | 0,029 | 0,019 | 0,027 | -0,037 | 0,032 | NA | NA |  |
| rs2532276 | A | C | -0,022 | 0,003 | -0,074 | 0,024 | -0,051 | 0,030 | -0,103 | 0,033 | -0,040 | 0,022 | -0,052 | 0,022 | -0,117 | 0,035 |  |
| rs2764771 | A | G | 0,010 | 0,002 | -0,037 | 0,018 | -0,089 | 0,023 | 0,015 | 0,025 | 0,004 | 0,017 | 0,001 | 0,018 | -0,010 | 0,026 |  |
| rs281379 | A | G | 0,014 | 0,001 | 0,067 | 0,017 | 0,019 | 0,021 | 0,140 | 0,024 | 0,002 | 0,017 | -0,059 | 0,017 | -0,113 | 0,030 |  |
| rs2854334 | G | A | 0,009 | 0,001 | -0,008 | 0,017 | 0,006 | 0,022 | -0,026 | 0,024 | -0,019 | 0,020 | -0,016 | 0,017 | -0,049 | 0,031 |  |
| rs28601761 | G | C | 0,009 | 0,001 | 0,012 | 0,017 | 0,009 | 0,022 | -0,003 | 0,024 | 0,014 | 0,016 | -0,022 | 0,017 | 0,030 | 0,026 |  |
| rs28680958 | A | G | -0,011 | 0,002 | -0,015 | 0,020 | -0,023 | 0,025 | 0,002 | 0,027 | 0,000 | 0,024 | 0,023 | 0,020 | -0,041 | 0,038 |  |
| rs28929474 | T | C | -0,037 | 0,005 | 0,157 | 0,064 | 0,195 | 0,079 | 0,118 | 0,088 | 0,002 | 0,056 | 0,101 | 0,060 | 0,174 | 0,118 |  |
| rs35034355 | A | G | -0,008 | 0,001 | 0,017 | 0,017 | 0,022 | 0,021 | 0,015 | 0,023 | 0,027 | 0,019 | -0,012 | 0,016 | 0,010 | 0,044 |  |
| rs35538052 | A | G | -0,009 | 0,002 | -0,030 | 0,018 | -0,025 | 0,022 | -0,014 | 0,024 | 0,006 | 0,020 | 0,005 | 0,017 | -0,030 | 0,029 |  |
| rs36052336 | G | A | -0,018 | 0,003 | -0,025 | 0,036 | -0,017 | 0,044 | -0,025 | 0,050 | 0,009 | 0,043 | 0,009 | 0,039 | 0,104 | 0,064 |  |
| rs3748034 | T | G | -0,012 | 0,002 | 0,001 | 0,027 | -0,009 | 0,033 | 0,016 | 0,037 | 0,025 | 0,035 | 0,036 | 0,026 | -0,010 | 0,066 |  |
| rs378421 | A | G | -0,011 | 0,001 | 0,090 | 0,019 | 0,052 | 0,023 | 0,149 | 0,025 | 0,050 | 0,026 | NA | NA | 0,010 | 0,029 |  |
| rs3803800 | G | A | 0,011 | 0,002 | 0,004 | 0,021 | -0,002 | 0,026 | -0,008 | 0,029 | 0,020 | 0,019 | -0,036 | 0,020 | 0,030 | 0,037 |  |
| rs3809162 | G | A | 0,009 | 0,001 | -0,017 | 0,017 | 0,006 | 0,022 | -0,052 | 0,024 | -0,007 | 0,021 | 0,001 | 0,016 | NA | NA |  |
| rs4092465 | G | A | -0,008 | 0,002 | -0,018 | 0,018 | -0,011 | 0,023 | -0,024 | 0,024 | -0,007 | 0,023 | -0,020 | 0,017 | -0,010 | 0,020 |  |
| rs4501255 | G | C | 0,011 | 0,002 | 0,031 | 0,020 | 0,031 | 0,025 | 0,048 | 0,027 | 0,023 | 0,023 | 0,009 | 0,019 | -0,041 | 0,035 |  |
| rs4548913 | A | G | -0,008 | 0,002 | 0,007 | 0,018 | 0,003 | 0,022 | 0,008 | 0,024 | -0,022 | 0,021 | -0,035 | 0,018 | 0,030 | 0,027 |  |
| rs4690727 | G | C | 0,011 | 0,002 | -0,013 | 0,019 | -0,018 | 0,024 | -0,006 | 0,025 | -0,076 | 0,022 | 0,002 | 0,018 | 0,030 | 0,024 |  |
| rs4699791 | A | G | 0,019 | 0,002 | 0,075 | 0,030 | 0,147 | 0,038 | 0,021 | 0,042 | 0,013 | 0,034 | -0,015 | 0,028 | 0,104 | 0,050 |  |
| rs4815364 | A | G | 0,009 | 0,001 | -0,022 | 0,018 | -0,026 | 0,023 | -0,010 | 0,024 | 0,013 | 0,021 | -0,017 | 0,017 | -0,010 | 0,033 |  |
| rs4842786 | A | G | -0,009 | 0,001 | 0,010 | 0,017 | 0,015 | 0,022 | -0,008 | 0,023 | -0,004 | 0,020 | -0,008 | 0,017 | -0,010 | 0,038 |  |
| rs4916723 | C | A | -0,010 | 0,001 | -0,005 | 0,017 | -0,027 | 0,022 | 0,025 | 0,024 | -0,040 | 0,020 | -0,019 | 0,017 | 0,030 | 0,025 |  |
| rs4938230 | A | C | 0,013 | 0,002 | -0,005 | 0,023 | 0,010 | 0,029 | -0,041 | 0,030 | -0,020 | 0,027 | 0,001 | 0,022 | -0,030 | 0,039 |  |
| rs500321 | T | A | -0,010 | 0,002 | -0,038 | 0,019 | -0,024 | 0,024 | -0,055 | 0,026 | NA | NA | NA | NA | 0,051 | 0,031 |  |
| rs5024204 | T | A | 0,010 | 0,002 | -0,023 | 0,019 | -0,017 | 0,024 | -0,035 | 0,026 | -0,003 | 0,022 | 0,007 | 0,018 | 0,030 | 0,030 |  |
| rs55872084 | T | G | 0,010 | 0,002 | -0,017 | 0,020 | -0,022 | 0,025 | -0,010 | 0,027 | -0,027 | 0,023 | -0,001 | 0,019 | -0,010 | 0,033 |  |
| rs55932213 | G | A | 0,009 | 0,002 | 0,009 | 0,020 | -0,010 | 0,025 | 0,056 | 0,027 | -0,013 | 0,024 | 0,008 | 0,021 | NA | NA |  |
| rs56030824 | A | G | -0,012 | 0,002 | 0,041 | 0,018 | 0,039 | 0,023 | 0,046 | 0,024 | 0,042 | 0,021 | -0,021 | 0,017 | -0,041 | 0,034 |  |
| rs561222871 | T | C | -0,039 | 0,004 | NA | NA | NA | NA | NA | NA | NA | NA | NA | NA | NA | NA |  |
| rs56337305 | C | T | -0,010 | 0,001 | -0,023 | 0,017 | -0,015 | 0,022 | -0,036 | 0,024 | -0,009 | 0,020 | 0,008 | 0,017 | -0,020 | 0,024 |  |
| rs58107686 | A | C | -0,010 | 0,002 | -0,058 | 0,018 | -0,081 | 0,023 | -0,036 | 0,024 | -0,004 | 0,021 | 0,058 | 0,017 | 0,010 | 0,035 |  |
| rs60654199 | A | C | -0,017 | 0,003 | -0,016 | 0,040 | 0,002 | 0,051 | -0,071 | 0,054 | 0,087 | 0,049 | 0,061 | 0,045 | 0,086 | 0,065 |  |
| rs62044525 | G | C | -0,012 | 0,002 | 0,017 | 0,022 | 0,026 | 0,027 | 0,001 | 0,029 | 0,015 | 0,020 | -0,021 | 0,021 | -0,010 | 0,033 |  |
| rs62250685 | G | A | -0,014 | 0,002 | 0,017 | 0,017 | 0,033 | 0,022 | -0,004 | 0,023 | -0,016 | 0,020 | -0,005 | 0,017 | 0,010 | 0,022 |  |
| rs6460047 | C | T | 0,012 | 0,002 | -0,042 | 0,022 | -0,013 | 0,027 | -0,061 | 0,030 | -0,037 | 0,029 | 0,035 | 0,024 | -0,051 | 0,038 |  |
| rs6787172 | G | T | -0,008 | 0,001 | 0,026 | 0,017 | 0,041 | 0,021 | 0,001 | 0,023 | -0,017 | 0,020 | 0,007 | 0,016 | NA | NA |  |
| rs682011 | C | T | 0,008 | 0,001 | -0,012 | 0,017 | -0,024 | 0,021 | 0,013 | 0,023 | -0,032 | 0,020 | -0,001 | 0,016 | 0,030 | 0,026 |  |
| rs6951574 | C | T | 0,013 | 0,001 | -0,004 | 0,020 | 0,002 | 0,024 | -0,003 | 0,029 | -0,027 | 0,022 | 0,005 | 0,019 | 0,030 | 0,027 |  |
| rs705687 | G | A | -0,011 | 0,002 | -0,015 | 0,021 | -0,012 | 0,026 | -0,021 | 0,028 | 0,005 | 0,024 | -0,027 | 0,020 | 0,020 | 0,043 |  |
| rs7074871 | A | G | -0,009 | 0,002 | -0,055 | 0,019 | -0,054 | 0,024 | -0,050 | 0,026 | 0,000 | 0,022 | 0,027 | 0,019 | -0,010 | 0,029 |  |
| rs7185555 | C | G | -0,011 | 0,002 | 0,030 | 0,024 | 0,055 | 0,030 | -0,007 | 0,033 | 0,028 | 0,027 | -0,039 | 0,024 | 0,010 | 0,028 |  |
| rs72859280 | T | G | 0,023 | 0,004 | -0,007 | 0,050 | -0,007 | 0,063 | 0,021 | 0,066 | -0,073 | 0,059 | 0,008 | 0,080 | 0,030 | 0,094 |  |
| rs74664784 | C | T | -0,013 | 0,002 | 0,020 | 0,017 | 0,033 | 0,022 | 0,000 | 0,023 | NA | NA | NA | NA | NA | NA |  |
| rs7682824 | T | C | 0,008 | 0,002 | NA | NA | NA | NA | NA | NA | NA | NA | NA | NA | NA | NA |  |
| rs77165542 | T | C | -0,026 | 0,004 | -0,012 | 0,055 | -0,024 | 0,066 | -0,009 | 0,079 | NA | NA | -0,523 | 0,307 | 0,039 | 0,082 |  |
| rs79139602 | T | A | 0,060 | 0,005 | -0,041 | 0,060 | -0,033 | 0,076 | -0,024 | 0,082 | -0,014 | 0,069 | -0,026 | 0,065 | 0,140 | 0,095 |  |
| rs7950166 | T | C | -0,010 | 0,002 | 0,007 | 0,017 | -0,011 | 0,022 | 0,031 | 0,024 | -0,005 | 0,020 | -0,022 | 0,017 | 0,010 | 0,030 |  |
| rs79616692 | C | G | 0,016 | 0,002 | 0,010 | 0,027 | 0,082 | 0,034 | -0,062 | 0,037 | -0,022 | 0,032 | -0,032 | 0,028 | 0,010 | 0,048 |  |
| rs823114 | A | G | 0,009 | 0,001 | 0,054 | 0,017 | 0,058 | 0,021 | 0,071 | 0,023 | 0,010 | 0,015 | -0,027 | 0,016 | 0,062 | 0,028 |  |
| rs828867 | A | G | 0,009 | 0,001 | -0,033 | 0,018 | -0,025 | 0,023 | -0,042 | 0,024 | 0,022 | 0,021 | 0,001 | 0,018 | 0,030 | 0,034 |  |
| rs9607814 | A | C | -0,010 | 0,002 | 0,026 | 0,021 | 0,009 | 0,026 | 0,057 | 0,028 | -0,028 | 0,025 | 0,018 | 0,021 | 0,030 | 0,028 |  |
| rs9838144 | C | G | -0,010 | 0,002 | -0,004 | 0,021 | -0,015 | 0,026 | 0,019 | 0,028 | 0,010 | 0,024 | -0,008 | 0,021 | NA | NA |  |
| rs9950000 | T | C | -0,009 | 0,001 | 0,060 | 0,017 | 0,062 | 0,022 | 0,052 | 0,023 | 0,028 | 0,016 | 0,037 | 0,017 | NA | NA |  |
| A1: effect allele; A2: other allele; se: standard errors; IBD: inflammatory bowel disease; UC: ulcerative colitis; CD: Crohn's disease; RA: rheumatoid arthritis; MS: multiple sclerosis; SLE: systemic lupus erythematosus. | | | | | | | | | | | | | | | | | |

| **Supplementary Table 5. Results from leave one out analysis which excludes one SNP at-a-time and performes analysis on the remaining SNPs.** | | | | | | | | | | | | |
| --- | --- | --- | --- | --- | --- | --- | --- | --- | --- | --- | --- | --- |
| SNP left out | IBD | | UC | | CD | | RA | | MS | | SLE | |
|  | OR | P-value | OR | P-value | OR | P-value | OR | P-value | OR | P-value | OR | P-value |
| rs10004020 | 0,84 | 0,44 | 0,93 | 0,77 | 0,71 | 0,26 | 0,79 | 0,24 | 0,74 | 0,16 | NA | NA |
| rs10028756 | 0,84 | 0,44 | 0,96 | 0,86 | 0,68 | 0,22 | 0,80 | 0,29 | 0,72 | 0,12 | 1,25 | 0,56 |
| rs1004787 | 0,85 | 0,46 | 0,95 | 0,83 | 0,70 | 0,26 | 0,78 | 0,21 | 0,75 | 0,17 | 1,14 | 0,74 |
| rs10085696 | 0,83 | 0,40 | 0,93 | 0,76 | 0,68 | 0,22 | 0,80 | 0,26 | 0,75 | 0,17 | 1,08 | 0,84 |
| rs10236149 | 0,84 | 0,44 | 0,94 | 0,80 | 0,70 | 0,25 | 0,82 | 0,33 | 0,76 | 0,19 | NA | NA |
| rs10438820 | 0,83 | 0,41 | 0,93 | 0,76 | 0,69 | 0,23 | 0,79 | 0,26 | 0,76 | 0,19 | 1,09 | 0,83 |
| rs10506274 | 0,83 | 0,40 | 0,93 | 0,76 | 0,68 | 0,21 | 0,82 | 0,33 | 0,74 | 0,16 | NA | NA |
| rs10750025 | 0,82 | 0,38 | 0,92 | 0,72 | 0,69 | 0,24 | 0,79 | 0,26 | 0,73 | 0,13 | 1,08 | 0,84 |
| rs10753661 | 0,84 | 0,43 | 0,94 | 0,78 | 0,70 | 0,25 | 0,80 | 0,27 | 0,73 | 0,14 | NA | NA |
| rs10876188 | 0,85 | 0,46 | 0,94 | 0,80 | 0,71 | 0,27 | 0,79 | 0,24 | 0,77 | 0,21 | 1,11 | 0,79 |
| rs10978550 | 0,83 | 0,41 | 0,94 | 0,80 | 0,68 | 0,22 | 0,82 | 0,32 | 0,74 | 0,16 | 1,17 | 0,69 |
| rs11030084 | 0,82 | 0,37 | 0,90 | 0,67 | 0,70 | 0,24 | 0,80 | 0,28 | 0,74 | 0,15 | NA | NA |
| rs1104608 | 0,85 | 0,45 | 0,94 | 0,81 | 0,71 | 0,26 | 0,79 | 0,26 | 0,75 | 0,18 | 1,16 | 0,70 |
| rs1123285 | 0,82 | 0,37 | 0,91 | 0,69 | 0,69 | 0,23 | 0,79 | 0,24 | 0,74 | 0,15 | 1,15 | 0,73 |
| rs113443718 | 0,84 | 0,44 | 0,95 | 0,82 | 0,70 | 0,25 | 0,82 | 0,32 | 0,76 | 0,18 | 1,03 | 0,94 |
| rs1154414 | 0,84 | 0,44 | 0,93 | 0,75 | 0,72 | 0,29 | 0,80 | 0,27 | 0,73 | 0,13 | 0,97 | 0,93 |
| rs11625650 | 0,84 | 0,42 | 0,92 | 0,73 | 0,70 | 0,25 | 0,81 | 0,29 | 0,74 | 0,16 | 1,09 | 0,83 |
| rs11692435 | 0,83 | 0,39 | 0,92 | 0,74 | 0,69 | 0,23 | 0,81 | 0,30 | 0,75 | 0,16 | 1,10 | 0,82 |
| rs11739827 | 0,85 | 0,45 | 0,95 | 0,82 | 0,70 | 0,25 | 0,79 | 0,24 | 0,74 | 0,16 | 1,07 | 0,86 |
| rs11940694 | 0,79 | 0,31 | 0,88 | 0,61 | 0,68 | 0,23 | 0,82 | 0,33 | 0,75 | 0,17 | 1,04 | 0,92 |
| rs12088813 | 0,83 | 0,41 | 0,93 | 0,77 | 0,69 | 0,23 | 0,79 | 0,24 | 0,74 | 0,15 | 1,12 | 0,77 |
| rs1217091 | 0,83 | 0,40 | 0,93 | 0,76 | 0,69 | 0,23 | 0,81 | 0,30 | 0,75 | 0,18 | 1,06 | 0,88 |
| rs1229984 | 0,81 | 0,42 | 0,92 | 0,76 | 0,60 | 0,17 | 0,71 | 0,12 | 0,70 | 0,11 | NA | NA |
| rs12499107 | 0,85 | 0,47 | 0,95 | 0,83 | 0,71 | 0,26 | 0,81 | 0,30 | 0,73 | 0,14 | 1,09 | 0,83 |
| rs1260326 | 0,92 | 0,70 | 1,03 | 0,92 | 0,78 | 0,41 | 0,75 | 0,17 | 0,72 | 0,13 | 1,23 | 0,60 |
| rs12651313 | 0,84 | 0,44 | 0,94 | 0,80 | 0,70 | 0,25 | 0,80 | 0,27 | 0,74 | 0,15 | 1,15 | 0,72 |
| rs12655091 | 0,84 | 0,42 | 0,92 | 0,73 | 0,71 | 0,26 | 0,80 | 0,26 | 0,77 | 0,20 | 1,13 | 0,76 |
| rs12795042 | 0,83 | 0,41 | 0,93 | 0,76 | 0,70 | 0,25 | 0,81 | 0,30 | 0,75 | 0,17 | NA | NA |
| rs12907323 | 0,83 | 0,40 | 0,93 | 0,76 | 0,68 | 0,22 | 0,83 | 0,35 | 0,75 | 0,17 | 1,15 | 0,73 |
| rs13024996 | 0,86 | 0,49 | 0,95 | 0,81 | 0,71 | 0,27 | 0,79 | 0,24 | 0,75 | 0,17 | 1,10 | 0,81 |
| rs13032049 | 0,84 | 0,43 | 0,95 | 0,82 | 0,69 | 0,23 | 0,83 | 0,34 | 0,76 | 0,19 | 1,17 | 0,69 |
| rs13066454 | 0,84 | 0,43 | 0,94 | 0,78 | 0,70 | 0,24 | 0,80 | 0,27 | 0,74 | 0,15 | 1,13 | 0,75 |
| rs13094887 | 0,85 | 0,46 | 0,94 | 0,81 | 0,71 | 0,27 | 0,82 | 0,32 | 0,77 | 0,20 | NA | NA |
| rs13107325 | 0,90 | 0,61 | 0,95 | 0,83 | 0,80 | 0,44 | 0,78 | 0,23 | 0,79 | 0,25 | 1,00 | 1,00 |
| rs13250583 | 0,85 | 0,47 | 0,95 | 0,81 | 0,71 | 0,27 | 0,80 | 0,28 | 0,77 | 0,21 | 1,12 | 0,77 |
| rs13383034 | 0,86 | 0,51 | 0,96 | 0,86 | 0,74 | 0,32 | 0,76 | 0,17 | 0,74 | 0,16 | 1,15 | 0,73 |
| rs144198753 | 0,83 | 0,41 | 0,94 | 0,80 | 0,68 | 0,21 | NA | NA | NA | NA | 1,05 | 0,90 |
| rs17029090 | 0,84 | 0,42 | 0,94 | 0,79 | 0,69 | 0,24 | 0,79 | 0,26 | 0,73 | 0,14 | 1,15 | 0,72 |
| rs1713676 | 0,84 | 0,42 | 0,94 | 0,78 | 0,69 | 0,24 | 0,81 | 0,29 | 0,75 | 0,18 | 1,10 | 0,81 |
| rs17177078 | 0,81 | 0,33 | 0,91 | 0,70 | 0,66 | 0,18 | 0,80 | 0,29 | 0,74 | 0,16 | 1,09 | 0,83 |
| rs17665139 | 0,84 | 0,43 | 0,93 | 0,77 | 0,69 | 0,24 | 0,81 | 0,29 | 0,74 | 0,15 | 1,06 | 0,87 |
| rs2011092 | 0,85 | 0,47 | 0,93 | 0,76 | 0,72 | 0,29 | 0,82 | 0,32 | 0,71 | 0,09 | 1,08 | 0,84 |
| rs2165670 | 0,82 | 0,39 | 0,92 | 0,72 | 0,69 | 0,24 | 0,78 | 0,23 | 0,73 | 0,13 | 1,18 | 0,67 |
| rs2178197 | 0,83 | 0,39 | 0,90 | 0,65 | 0,70 | 0,26 | 0,81 | 0,29 | 0,76 | 0,19 | 1,04 | 0,93 |
| rs2180870 | 0,85 | 0,47 | 0,94 | 0,79 | 0,72 | 0,28 | 0,82 | 0,32 | 0,75 | 0,18 | 1,13 | 0,75 |
| rs2472297 | 0,83 | 0,40 | 0,92 | 0,72 | 0,70 | 0,25 | 0,79 | 0,25 | 0,75 | 0,17 | NA | NA |
| rs2532276 | 0,79 | 0,28 | 0,90 | 0,65 | 0,64 | 0,15 | 0,75 | 0,15 | 0,69 | 0,07 | 0,93 | 0,84 |
| rs2764771 | 0,86 | 0,48 | 0,98 | 0,95 | 0,69 | 0,23 | 0,79 | 0,26 | 0,74 | 0,16 | 1,12 | 0,77 |
| rs281379 | 0,78 | 0,26 | 0,92 | 0,71 | 0,61 | 0,09 | 0,79 | 0,26 | 0,80 | 0,28 | 1,28 | 0,51 |
| rs2854334 | 0,84 | 0,44 | 0,93 | 0,76 | 0,71 | 0,26 | 0,81 | 0,30 | 0,75 | 0,18 | 1,15 | 0,72 |
| rs28601761 | 0,83 | 0,40 | 0,93 | 0,76 | 0,70 | 0,24 | 0,79 | 0,23 | 0,76 | 0,19 | 1,07 | 0,86 |
| rs28680958 | 0,83 | 0,40 | 0,92 | 0,73 | 0,70 | 0,24 | 0,80 | 0,27 | 0,76 | 0,18 | 1,08 | 0,85 |
| rs28929474 | 0,86 | 0,50 | 0,97 | 0,89 | 0,71 | 0,27 | 0,80 | 0,27 | 0,76 | 0,20 | 1,15 | 0,72 |
| rs35034355 | 0,85 | 0,45 | 0,95 | 0,82 | 0,70 | 0,25 | 0,81 | 0,31 | 0,74 | 0,15 | 1,11 | 0,79 |
| rs35538052 | 0,82 | 0,38 | 0,92 | 0,73 | 0,69 | 0,23 | 0,80 | 0,28 | 0,75 | 0,17 | 1,08 | 0,85 |
| rs36052336 | 0,83 | 0,40 | 0,93 | 0,76 | 0,69 | 0,23 | 0,80 | 0,28 | 0,75 | 0,16 | 1,15 | 0,72 |
| rs3748034 | 0,84 | 0,42 | 0,93 | 0,76 | 0,70 | 0,25 | 0,80 | 0,29 | 0,76 | 0,18 | 1,10 | 0,80 |
| rs378421 | 0,89 | 0,57 | 0,97 | 0,89 | 0,76 | 0,35 | 0,82 | 0,32 | NA | NA | 1,12 | 0,78 |
| rs3803800 | 0,84 | 0,42 | 0,93 | 0,78 | 0,70 | 0,25 | 0,78 | 0,22 | 0,76 | 0,20 | 1,08 | 0,84 |
| rs3809162 | 0,85 | 0,45 | 0,93 | 0,76 | 0,72 | 0,28 | 0,80 | 0,28 | 0,74 | 0,16 | NA | NA |
| rs4092465 | 0,83 | 0,40 | 0,93 | 0,76 | 0,69 | 0,23 | 0,80 | 0,26 | 0,73 | 0,14 | 1,09 | 0,83 |
| rs4501255 | 0,82 | 0,38 | 0,92 | 0,72 | 0,68 | 0,21 | 0,79 | 0,24 | 0,74 | 0,15 | 1,14 | 0,74 |
| rs4548913 | 0,84 | 0,43 | 0,93 | 0,78 | 0,70 | 0,25 | 0,79 | 0,24 | 0,73 | 0,12 | 1,14 | 0,74 |
| rs4690727 | 0,84 | 0,44 | 0,94 | 0,81 | 0,70 | 0,25 | 0,84 | 0,38 | 0,74 | 0,16 | 1,06 | 0,89 |
| rs4699791 | 0,81 | 0,34 | 0,88 | 0,56 | 0,69 | 0,23 | 0,79 | 0,25 | 0,75 | 0,17 | 1,04 | 0,93 |
| rs4815364 | 0,85 | 0,45 | 0,95 | 0,82 | 0,70 | 0,25 | 0,79 | 0,25 | 0,75 | 0,18 | 1,11 | 0,79 |
| rs4842786 | 0,84 | 0,44 | 0,94 | 0,80 | 0,69 | 0,24 | 0,80 | 0,26 | 0,74 | 0,15 | 1,10 | 0,81 |
| rs4916723 | 0,83 | 0,41 | 0,92 | 0,72 | 0,71 | 0,26 | 0,77 | 0,20 | 0,73 | 0,13 | 1,15 | 0,72 |
| rs4938230 | 0,84 | 0,43 | 0,93 | 0,76 | 0,71 | 0,27 | 0,81 | 0,29 | 0,74 | 0,16 | 1,13 | 0,76 |
| rs500321 | 0,82 | 0,37 | 0,92 | 0,73 | 0,68 | 0,20 | NA | NA | NA | NA | 1,15 | 0,71 |
| rs5024204 | 0,85 | 0,46 | 0,94 | 0,81 | 0,71 | 0,27 | 0,80 | 0,27 | 0,74 | 0,15 | 1,08 | 0,85 |
| rs55872084 | 0,84 | 0,44 | 0,94 | 0,81 | 0,70 | 0,25 | 0,81 | 0,30 | 0,74 | 0,16 | 1,11 | 0,78 |
| rs55932213 | 0,83 | 0,41 | 0,94 | 0,79 | 0,68 | 0,21 | 0,80 | 0,28 | 0,74 | 0,15 | NA | NA |
| rs56030824 | 0,86 | 0,50 | 0,96 | 0,87 | 0,72 | 0,28 | 0,83 | 0,34 | 0,73 | 0,13 | 1,07 | 0,87 |
| rs561222871 | 0,80 | 0,33 | 0,92 | 0,73 | 0,66 | 0,19 | NA | NA | NA | NA | NA | NA |
| rs56337305 | 0,82 | 0,38 | 0,92 | 0,74 | 0,68 | 0,21 | 0,79 | 0,25 | 0,75 | 0,17 | 1,08 | 0,85 |
| rs58107686 | 0,81 | 0,32 | 0,89 | 0,60 | 0,68 | 0,21 | 0,80 | 0,26 | 0,78 | 0,23 | 1,11 | 0,79 |
| rs60654199 | 0,83 | 0,41 | 0,93 | 0,78 | 0,69 | 0,22 | 0,81 | 0,31 | 0,75 | 0,18 | 1,14 | 0,74 |
| rs62044525 | 0,84 | 0,45 | 0,95 | 0,82 | 0,70 | 0,24 | 0,81 | 0,30 | 0,73 | 0,14 | 1,10 | 0,82 |
| rs62250685 | 0,85 | 0,46 | 0,96 | 0,88 | 0,69 | 0,24 | 0,78 | 0,23 | 0,74 | 0,15 | 1,14 | 0,75 |
| rs6460047 | 0,85 | 0,48 | 0,94 | 0,79 | 0,72 | 0,28 | 0,81 | 0,30 | 0,73 | 0,13 | 1,14 | 0,73 |
| rs6787172 | 0,85 | 0,46 | 0,96 | 0,85 | 0,70 | 0,24 | 0,79 | 0,24 | 0,75 | 0,17 | NA | NA |
| rs682011 | 0,84 | 0,44 | 0,95 | 0,82 | 0,69 | 0,23 | 0,82 | 0,31 | 0,74 | 0,16 | 1,07 | 0,86 |
| rs6951574 | 0,84 | 0,43 | 0,93 | 0,77 | 0,70 | 0,24 | 0,82 | 0,32 | 0,74 | 0,15 | 1,06 | 0,89 |
| rs705687 | 0,83 | 0,40 | 0,93 | 0,75 | 0,69 | 0,23 | 0,80 | 0,28 | 0,73 | 0,13 | 1,12 | 0,78 |
| rs7074871 | 0,81 | 0,34 | 0,91 | 0,68 | 0,68 | 0,21 | 0,80 | 0,27 | 0,76 | 0,19 | 1,10 | 0,82 |
| rs7185555 | 0,85 | 0,45 | 0,95 | 0,85 | 0,69 | 0,24 | 0,81 | 0,30 | 0,73 | 0,13 | 1,12 | 0,78 |
| rs72859280 | 0,84 | 0,43 | 0,93 | 0,78 | 0,69 | 0,24 | 0,81 | 0,30 | 0,74 | 0,16 | 1,10 | 0,81 |
| rs74664784 | 0,85 | 0,47 | 0,96 | 0,87 | 0,69 | 0,24 | NA | NA | NA | NA | NA | NA |
| rs7682824 | NA | NA | NA | NA | NA | NA | NA | NA | NA | NA | NA | NA |
| rs77165542 | 0,84 | 0,42 | 0,93 | 0,76 | 0,70 | 0,24 | NA | NA | 0,74 | 0,15 | 1,12 | 0,77 |
| rs79139602 | 0,85 | 0,46 | 0,94 | 0,81 | 0,70 | 0,25 | 0,80 | 0,28 | 0,75 | 0,17 | 1,02 | 0,96 |
| rs7950166 | 0,84 | 0,43 | 0,93 | 0,75 | 0,71 | 0,27 | 0,80 | 0,26 | 0,73 | 0,13 | 1,12 | 0,78 |
| rs79616692 | 0,83 | 0,41 | 0,90 | 0,65 | 0,71 | 0,27 | 0,81 | 0,29 | 0,76 | 0,18 | 1,10 | 0,81 |
| rs823114 | 0,81 | 0,33 | 0,90 | 0,65 | 0,67 | 0,18 | 0,79 | 0,24 | 0,76 | 0,19 | 1,04 | 0,91 |
| rs828867 | 0,85 | 0,47 | 0,95 | 0,82 | 0,71 | 0,27 | 0,79 | 0,24 | 0,74 | 0,16 | 1,08 | 0,84 |
| rs9607814 | 0,85 | 0,46 | 0,94 | 0,79 | 0,71 | 0,28 | 0,79 | 0,24 | 0,75 | 0,18 | 1,14 | 0,73 |
| rs9838144 | 0,84 | 0,42 | 0,93 | 0,75 | 0,70 | 0,25 | 0,80 | 0,28 | 0,74 | 0,15 | NA | NA |
| rs9950000 | 0,87 | 0,52 | 0,97 | 0,90 | 0,72 | 0,28 | 0,82 | 0,34 | 0,77 | 0,20 | NA | NA |
| OR: odds ratio; IBD: inflammatory bowel disease; UC: ulcerative colitis; CD: Crohn's disease; RA: rheumatoid arthritis; MS: multiple sclerosis; SLE: systemic lupus erythematosus | | | | | | | | | | | | |

**Supplementary Figure 1. Flowchart of our Mendelian randomization study on alcohol consumption and four autoimmune disorders.**

Primary statistical analysis for causal relationship

- a random-effect inverse variance-weighted method (IVW)
- a maximum likelihood approach
- a weighted-median approach
- an MR-Egger regression

Outcome: four autoimmune inflammatory disorders

- Inflammatory bowel disease: 12,882/21,770
- Ulcerative colitis: 6,968/20,464
- Crohn’s disease: 5,956/14,927
- Rheumatoid arthritis: 14,361/43,923
- Multiple sclerosis: 14,802/26,703
- Systemic lupus: 7,291/15,991

Exposure 1. GWAS of alcohol consumption on 941,280 individuals of European ancestry

- 99 biallelic common genetic variants (SNPs) associated with drinks/ week

Exposure 2. GWAS of alcohol use disorder on 274,424 veterans of European ancestry

- 13 biallelic common genetic variants associated with excessive drinking
- Genetic predictors for drinks/week
- Genetic predictors for excessive drinking

Confounding and pleiotropic effect was detected and controlled by several sensitivity analysis

- MR-Egger intercept test
- Excluding palindromic SNPs
- Excluding pleiotropic SNPs
- Multivariable MR adjusting for the effect of smoking and obesity
